# Cas9-directed long-read sequencing to resolve optical genome mapping findings in leukemia diagnostics

**DOI:** 10.1038/s41598-024-59092-6

**Published:** 2024-04-12

**Authors:** Eddy N. de Boer, Vincent Vroom, Arjen J. Scheper, Lennart F. Johansson, Laura Bosscher, Nettie Rietema, Sabrina Z. Commandeur-Jan, Nine V. A. M. Knoers, Birgit Sikkema-Raddatz, Eva van den Berg, Cleo C. van Diemen

**Affiliations:** Department of Genetics, University of Groningen, University Medical Center Groningen, CB51, Hanzeplein 1, 9713 GZ Groningen, The Netherlands

**Keywords:** Biological techniques, Cancer, Genetics, Health care, Medical research, Oncology

## Abstract

Leukemias are genetically heterogeneous and diagnostics therefore includes various standard-of-care (SOC) techniques, including karyotyping, SNP-array and FISH. Optical genome mapping (OGM) may replace these as it detects different types of structural aberrations simultaneously and additionally detects much smaller aberrations (500 bp vs 5–10 Mb with karyotyping). However, its resolution may still be too low to define clinical relevance of aberrations when they are located between two OGM labels or when labels are not distinct enough. Here, we test the potential of Cas9-directed long-read sequencing (LRS) as an additional technique to resolve such potentially relevant new findings. From an internal Bionano implementation study we selected ten OGM calls that could not be validated with SOC methods. Per variant we designed crRNAs for Cas9 enrichment, prepared libraries and sequenced them on a MinION/GridION device. We could confirm all aberrations and, importantly, the actual breakpoints of the OGM calls were located between 0.2 and 5.5 kb of the OGM-estimated breakpoints, confirming the high reliability of OGM. Furthermore, we show examples of redefinition of aberrations between labels that enable judgment of clinical relevance. Our results suggest that Cas9-directed LRS can be a relevant and flexible secondary technique in diagnostic workflows including OGM.

## Introduction

Leukemias are clinically and genetically heterogeneous cancers of the white blood cells that originate in the bone marrow. They are caused by somatic genetic aberrations, including single nucleotide variants (SNVs), small indels, aneuploidies, copy number variations (CNVs), inversions, insertions and (complex) chromosomal rearrangements^1–3^. Classification of the different types of hematologic malignancies in leukemia is based on the morphology of the cells, immunophenotypic profiles and the identification of specific (cyto)genetic abnormalities^4^. As the type of genetic aberration(s) a patient carries in their bone marrow is one of the factors that determine progression prognosis and choice of treatment^5^ a rapid and comprehensive genetic diagnosis is critical.

Optical genome mapping (OGM) (Bionano Genomics, San Diego, CA) is an emerging technique that has the potential to detect structural aberrations in different types of leukemia^6^. OGM images long (on average 300 kb), directly labeled fragments of genomic DNA. Based on their labels, these images are either directly aligned to a reference genome or de novo-assembled before alignment. Several studies using OGM have confirmed that it can identify a wide variety of previously detected aberrations in unselected cohorts of patients with various types of hematologic malignancies (e.g.,^7,8^).

Although OGM can detect aberrations up to 500 bp with de novo*-*assembly (Bionano, 30,110 Rev K), the resolution may be too low as OGM is not able to define the exact location of deletions and duplications that fall between two labels and OGM breakpoints are disputable when the label pattern is not distinctive enough. The exact characterization of aberrations can, however, have significant clinical relevance, for example if an OGM-deleted region between two labels contains a relevant gene and lack of resolution makes it uncertain whether the gene is affected. The current standard-of-care (SOC) methods to validate OGM findings also have limitations. Karyotyping has a limited resolution of 5–10 Mb and requires culturing whereas SNP-array has a resolution of 150 kb and can only detect unbalanced aberrations.

In this proof-of-principle study, we tested whether Cas9-directed long-read sequencing (LRS) can be used to characterize aberrations identified with OGM when single-basepair-level resolution is required and/or the resolution of SOC methods is insufficient to confirm OGM detected aberrations. The procedure was used for different types of aberrations in leukemia samples.

## Methods

### Sample selection and workflow

For an internal OGM implementation study, we prospectively collected 18 bone marrow aspirates (BMA) taken from patients with different reasons for referral, a collection set up with a maximum chance of detection of diverse types of aberrations. The inclusion criterion was sufficient BMA to perform OGM alongside current diagnostics. OGM and follow-up experiments were performed in all samples in accordance with the regulations and ethical guidelines of the UMCG as approved by the medical ethics review board from the UMCG (M23.321720). All patients gave informed consent for use of diagnostically obtained materials for innovations of diagnostic care and the study protocol was approved by certified laboratory specialists.

OGM aberrations were detected as described in Table 1 and Supplemental Information 3. The OGM identified aberrations were then compared to the variants identified using the SOC diagnostic methods. When possible, additional OGM findings were confirmed with the SOC methods karyotyping and the Infinium Global Screening Array-24 v3.0-EA-MD (SNP-Array) (Illumina, San Diego, CA), according to standard protocols. For this proof-of-principle study, we selected samples for Cas9-directed LRS (Oxford Nanopore Technologies, Oxford, UK) when we needed to redefine the breakpoints to improve characterization of the aberration or confirm additional findings that could not be detected with the SOC methods (Table 1).

**Table 1:** Selection of OGM findings for Cas9-directed long-read sequencing.

ID	Referral reason	OGM (splitted per aberration)	SOC	Identified with SOC	Detection problem
BMA1	AML/MDS/MPN	ogm[GRCh37]1q21.1q21.1(144452084_249237532) × 2 ~ 3	Karyotyping	Yes	
BMA2	CML	ogm[GRCh37]t(9;22)(q34;q11.2)(133619362_23603312)	Karyotyping	Yes	
BMA8	CML	ogm[GRCh37]t(9;22)(q34;q11.2)	Karyotyping	Yes	
BMA16	HCL (CLL)	ogm[GRCh37](X) × 1,(1–22) × 2	Karyotyping	Yes	
BMA16	HCL (CLL)	ogm[GRCh37]t(8;8)(p22;q13.2)(15.548.752;69.835.790)	Karyotyping (1:250 metaphases)	Yes	
BMA15	MDS/AML	ogm[GRCh37]t(5;5)(q14.2;q33.3)(81.692.066;159.453.630)	Karyotyping, SNP-arrays	Yes	
BMA8	CML	ogm[GRCh37]22q11.23;q12.1)(23,734,214;29,424,129) × 1	SNP-arrays	Yes	
BMA9	MDS	ogm[GRCh37]chr21 × 3	SNP-arrays	Yes	
BMA10	MDS/MPN	ogm[GRCh37]9p24.3p13.3(1_35889394) × 2 hmz	SNP-arrays	Yes	
BMA10	MDS/MPN	ogm[GRCh37]14q11.2q12(20421719_32361050) × 1	SNP-arrays	Yes	
BMA12	(B)-CLL	ogm[GRCh37]chr12 × 3	SNP-arrays	Yes	
BMA13	MDS	ogm[GRCh37] 20q11.22q13.13(34259243_49502193) × 1	SNP-arrays	Yes	
BMA16	HCL (CLL)	ogm[GRCh37]8p23.3p22(11805_15540773) × 1 ~ 2	SNP-arrays	Yes	
BMA1	AML/MDS/MPN	ogm[GRCh37]17p13.1(7,545,377_7,588,071) × 1	Could not be confirmed	No	Aberration located in between 2 OGM labels
BMA3	AML/MDS	ogm[GRCh37]17p13.1(7,545,377_7,588,071) × 1	Could not be confirmed	No	Aberration located in between 2 OGM labels
BMA6	MDS	ogm[GRCh37]17p13.1(7,545,377_7,588,071) × 1	Could not be confirmed	No	Aberration located in between 2 OGM labels
BMA17	MDS	ogm[GRCh37]ins(12p13.2)(11,889,007_11,895,594)	Could not be confirmed	No	Aberration located in between 2 OGM labels
BMA16	HCL (CLL)	ogm[GRCh37]t(14;17)(q32.22;q25.3) (106,249,815;80,915,618)	Could not be confirmed	No	Disputable breakpoint
BMA7	MDS/MF	ogm[GRCh37]inv(3)(q25.33q26.2)(160,014,744_168,882,939)	Could not be confirmed	No	Resolution SOC too low
BMA7	MDS/MF	ogm[GRCh37]3q25.33(159,902,689–160,014,744) × 1	Could not be confirmed	No	Resolution SOC too low
BMA7	MDS/MF	ogm[GRCh37]3q26.2(168,882,939–168,907,480) × 1	Could not be confirmed	No	Resolution SOC too low
BMA9	MDS	ogm[GRCh37]inv(15)(q24.1q24.1)(72,959,741_74,362,190)	Could not be confirmed	No	Resolution SOC too low
BMA9	MDS	ogm[GRCh37]3p13(71086423_71375386) × 1	Could not be confirmed	No	Resolution SOC too low

Aberrations detected using optical genome mapping (OGM) in a selected group of 18 bone marrow aspirates (BMA) with varying reasons for referral are depicted in this table. The table indicates if these aberrations were confirmed with the standard-of-care (SOC) methods (yes, no) and, if not, the detection problem is mentioned. Abbreviations referral reason: AML, acute myeloid leukemia; MDS, myelodysplastic syndrome; MPN, myeloproliferative neoplasms; CML, chronic myeloid leukemia; HCL, hairy cell leukemia; CLL, chronic lymphoblastic leukemia; (B)CLL, B-cell acute lymphoblastic leukemia; MF, myelofibrosis.

### DNA extraction, qualification and quantification

The input material for Cas9-directed LRS was the remaining Bionano-isolated ultra-high molecular weight DNA or Maxwell RSC (AS1400) (Promega, Madison, WI) or Nanobind CBB (Pacific Biosciences, Menlo Park, CA) extracted DNA. DNA length, quantity and purity were checked with FemtoPulse pulsed-field capillary electrophoresis (> 30 kb) (Agilent, Santa Clara, CA), Nanodrop spectrophotometry (OD 260/280 ~ 1.8, OD 260/230 2.0–2.2) (ThermoFisher Scientific, Waltham, MA) and Qubit (ThermoFisher).

### crRNA design for Cas9 enrichment

We used the CHOPCHOP web browser^9–11^ to search for candidate crRNA sequences. The ONT instructions (targeted, amplification-free DNA sequencing using Crispr/CAS, version ECI_S1014_v1_revE_11Dec2018) were used in combination with Genome build GRCh37. Efficiency scores of crRNAs were calculated according to “Doench et al. 2014—only for NGG PAM”^12^. The downloaded results were filtered as recommended by ONT: GC: 40–80%, self-complementarity score 0, retain efficiency score > 0.3 (Cas9 only), retain candidates with mismatches MM0 = 0, MM1 = 0, MM2 = 0, MM3 least possible. The crRNAs we designed are listed in Supplemental Information 4 and were ordered form IDT (IDT, Coralville, IA (IDT) as Alt-R™ Cas9 crRNAs.

### Enrichment, sample-preparation and sequencing

We used the ligation sequencing Cas9 enrichment protocol (SQK-CS9109, ONT) to enrich the regions of interest and prepare the samples for sequencing, following the manufacturer’s instructions. The protocol was started with an input of 1–5 µg HMW DNA. The library was purified with AMPure XP beads (A63881) (Beckman Coulter™, Fullerton, CA). An R9 MinION flowcell (FLO-MIN114) was primed, loaded and run on a MinION / GridION device, following ONT instructions.

### Cas9 data analysis

Basecalling was performed using MinKNOW v22.10.5 (ONT). Fastq files passing quality metrics were merged into a single fastq file and aligned with minimap2 v2.24^13^. Sniffles2 v2.0.7^14^ was used for SV calling. In addition, bam-files were viewed within IGV-viewer v2.14.1^15^. In association with a tumor cytogeneticist, we assessed the potential pathogenicity of the variants based on the literature and the WHO classification of tumors of hematopoietic and lymphoid tissues^4,16^.

### Ethical declarations

The authors have no conflicts of interest, including specific financial interests and relationships and affiliations relevant to the subject of the manuscript.

## Results

### Selection of samples for Cas9-directed long-read sequencing

The implementation study resulted in detection of 23 aberrations with OGM. Of these, 13 were confirmed with the results of the SOC methods, some performed as part of the diagnostic process. For these variants, it was not necessary to know the exact location of the breakpoints, and no Cas9-directed LRS confirmation was performed. This left 10 aberrations that could not be confirmed using the SOC methods and were selected to follow up with Cas9-directed LRS (Table 1).

### Confirmation of additional OGM findings and redefinition of the breakpoints with Cas9-directed long-read sequencing

We could detect each selected aberration using Cas9-directed LRS. We used at the maximum one R9 MinION flowcell (FLO-MIN114) per aberration, resulting in 1–25 LRS reads covering the aberration (Table 2).

**Table 2:** Summary of quality control measures of OGM and ONT.

BMA	Aberration OGM	Self-molecules	V.A.F	Number of reads crossing junction
Number		OGM	OGM##	ONT
BMA1	ogm[GRCh37]17p13.1(7,545,377_7,588,071) × 1	48	–	10
BMA3	ogm[GRCh37]17p13.1(7,545,377_7,588,071) × 1	67	–	1
BMA6	ogm[GRCh37]17p13.1(7,545,377_7,588,071) × 1	54	–	4
BMA17	ogm[GRCh37]ins(12p13.2)(11,889,007_11,895,594)	62	0.50	25
BMA16	ogm[GRCh37]t(14;17)(q32.22;q25.3)(106,249,815;80,915,618)	17	0.36	4
BMA7	ogm[GRCh37]inv(3)(q25.33q26.2)(160,014,744_168,882,939)	50	–	6, 19
BMA7	ogm[GRCh37]3q25.33(159,902,689–160,014,744) × 1	50	–	6
BMA7	ogm[GRCh37]3q26.2(168,882,939–168,907,480) × 1	50	–	19
BMA9	ogm[GRCh37]3p13(71,086,423_71,375,386) × 1	7	0.03	1
BMA9	ogm[GRCh37]inv(15)(q24.1q24.1)(72,959,741_74,362,190)**#**	48	0.32	R.O. 91, F.O. 17**###**

For each OGM aberration, OGM self-molecules, OGM variant allele frequency’s and ONT reads crossing the junction are depicted in this QC summary.

BMA, bone marrow aspirates; VAF, variant allele frequency; OGM, optical genome mapping; ONT, Oxford Nanopore Technologies; RO, reverse orientated; FO, forward orientated.

^#^ONT redefined OGM-call.

^##^Version 1.6 Access software, no V.A.F. information.

^###^Reverse and forward orientated adapter, not crossing junctions.

#### Redefinition of the breakpoints

**Figure 1:**
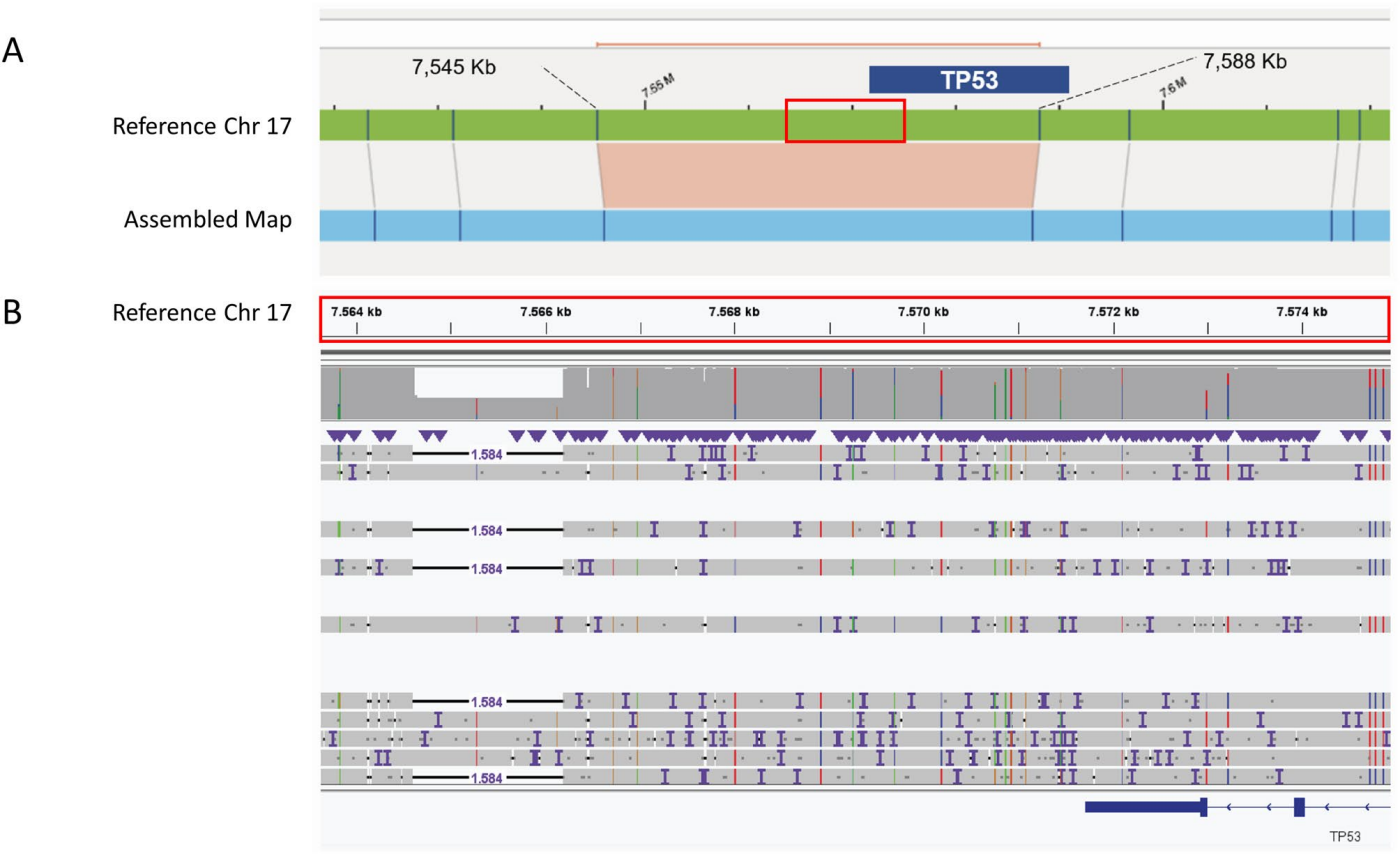
Representative example of an OGM aberration located between two OGM labels. (**A**) In three patients, OGM detected a heterozygous 1.2 kb deletion in a 42 kb region (17p13.1) between two OGM labels that overlaps with *TP53*. (**B**) Cas9-directed long-read sequencing redefined the aberration to a 1.6 kb deletion localized upstream of *TP53.* This aberration is a known CNV in the general population.

In three patients (BMA1, BMA3 and BMA6), OGM detected a heterozygous 1.2 kb deletion in a 42 kb region located between two OGM labels (ogm[GRCh37]17p13.1(7,545,377_7,588,071) × 1) that includes a part of *TP53* (chr17:7,571,720–7,590,868). Cas9-directed LRS redefined the deletion to a 1.6 kb (chr17:7,564,612–7,566,197) deletion localized adjacent to *TP53* (Fig. 1). Because *TP53* is not included in the deletion and it is also a known CNV in the general population, this aberration is not considered clinically relevant^17^.

In patient BMA17, referred for myelodysplastic syndrome, OGM detected a heterozygous 1.4 kb insertion in a 6.6 kb region located between two OGM labels (ogm[GRCh37]ins(12p13.2)(11,889,007_11,895,594)) that includes *ETV6* (chr12:11,802,788–12,048,325). Cas9-directed LRS redefined the insertion to 1.4 kb (supplementary information 5, Fig. 2). This 1.4 kb insertion is 99% similar to region chr12:11,892,052–11,893,440 (https://genome.ucsc.edu/cgi-bin/hgBlat) and is inserted in intron 1 (chr12:11,892,830–11,894,194) of the NM_001987 transcript of *ETV6*. The localization of this inserted region is deep intronic, and thus it probably has no clinical significance.

**Figure 2:**
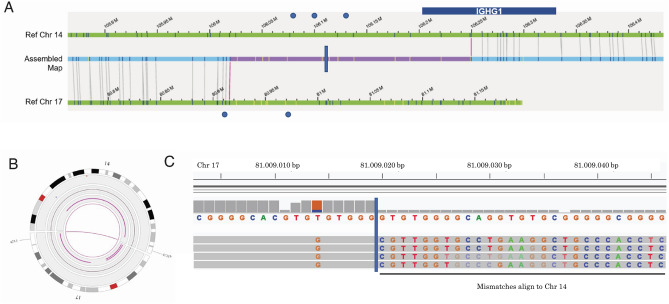
Representative example of an OGM aberration with a disputable breakpoint. OGM detected a translocation between chromosome 14 and 17 (q32.33; q25.3) that includes part of *IGHG1* (**A** & **B**). We evaluated the uncertain breakpoint region (purple area, **A**) and used this information to design crisprs (blue dots, **A**). Cas9-directed long-read sequencing redefined the breakpoints (blue vertical line, **A** & **C**). The chromosome 14 breakpoint is located 88 kb downstream of *IGHG1* (**A** & **C**).

In patient BMA16, referred for hairy cell leukemia, OGM detected a balanced translocation between chromosomes 14 and 17 (ogm[GRCh37]t(14;17)(q32.22;q2106,249,815;80,915,618)) that includes *IGHG1* with a disputable breakpoint (Fig. 2). This translocation was not seen in the original karyotyping using cultured cells, probably because mature aberrant cells were lost. The balanced translocation cannot be confirmed with SNP-array, and metaphase FISH would have the same limitation as karyotyping. Interphase FISH is no SOC in our laboratory for this particular region and was therefore not done. Cas9-directed LRS redefined the breakpoints to (chr14:106,114,465; chr17:81,009,018) (Fig. 2). The breakpoint on chromosome 14 (106,114,465) is located 88 kb downstream of *IGHG1* (chr14:106,202,680–106,209,408) (*IGHG1* is located on the reverse strand). This translocation has not been described in the context of leukemia.

#### Increased resolution required for confirmation of an OGM call

**Figure 3:**
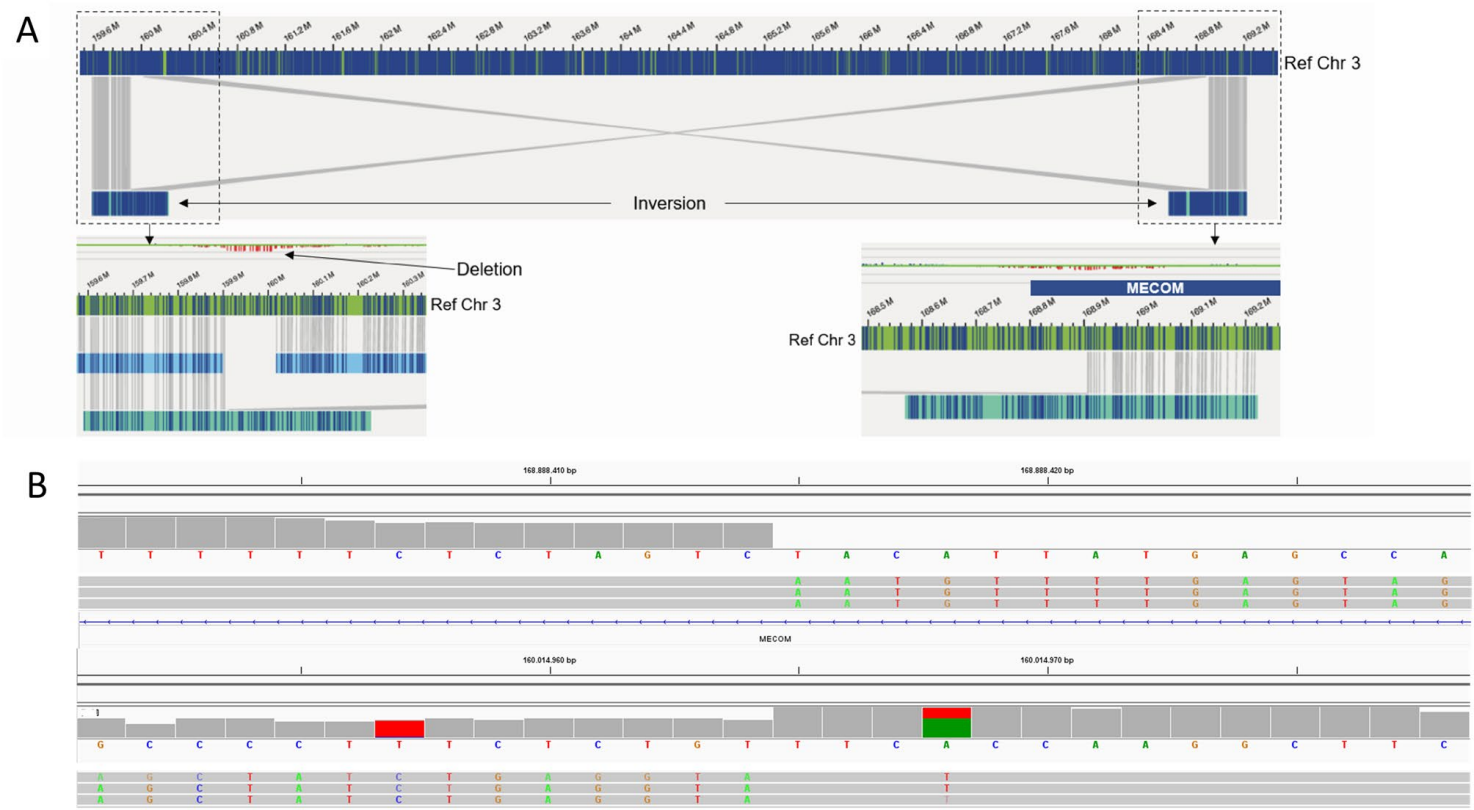
Representative example of finding where SOC resolution is too low. (**A**) OGM detected an 8.9 Mb inversion of chromosome 3(q25.33q26.2) that includes *MECOM*, while two adjacent 112 kb (q25.33) and 25 kb (q26.2) regions were deleted. (**B**) Cas9-directed long-read sequencing redefined the inversion to 8.9 Mb (160,014,965–168,888,415) and the deletions to 109 kb (159,905,686–160,014,965) and 19 kb (168,888,415–168,907,304). The inversion includes part of (NM-004991.3) or all of the *MECOM* transcript.

In patient BMA7, who was referred for myelodysplastic syndrome/myelofibrosis, OGM detected three aberrations: an 8.9 Mb inversion (ogm[GRCh37]inv(3)(q25.33q26.2)(160,014,744_168,882,939)), a 112 kb deletion at 3q25.33 (ogm[GRCh37]3q25.33(159,902,689–160,014,744) × 1) and a 25 kb deletion at 3q26.2 (ogm[GRCh37]3q26.2(168,882,939–168,907,480) × 1). Cas9-directed LRS redefined the inversion to 8.9 Mb (160,014,965–168,888,415) and the deletions to 109 kb (159,905,686–160,014,965) and 19 kb (168,888,415–168,907,304), respectively (Fig. 3). The redefined breakpoints are located, respectively 0.22, 5.5, 3.0, 0.22, 5.5 and 0.18 kb from the OGM breakpoints. The 19 kb deletion (168,888,415–168,907,304) is part of intron 2 of the NM-004991.3 transcript of *MECOM* and is located 5’ upstream of other transcripts. The 8.9 Mb inversion (160,014,965–168,888,415) includes part of transcript NM-004991.3 (intron 2 till exon 16), which will cause a non-functioning transcript. In the other transcripts, the inversion includes all of *MECOM*. However, this is not the previously identified inversion 3 (q21q26), resulting in *MECOM* expression being effected by the *GATA2* enhancer, that is associated with leukemia^18^.

In patient BMA9, who was referred for myelodysplastic syndrome, OGM detected a heterozygous 289 kb deletion that includes *FOXP1* (ogm[GRCh37]3p13(71,086,423_71,375,386) × 1). This aberration, which has an allele frequency of 3%, was only detected with the OGM rare variant pipeline (Supplemental Information 3). Cas9-directed LRS redefined the aberration to a 283 kb (71,087,137–71,370,212) deletion of intron 7 through intron 11 of *FOXP1* (supplementary information 5, Fig. 3). The redefined breakpoints are located 0.7 kb and 5 kb of the OGM breakpoints, respectively. *FOXP1* deletions have been described in acute myeloid leukemia and myelodysplastic syndrome, but the evidence for their clinical relevance is currently not strong^19,20^.

Finally, in patient BMA9, OGM detected a 305 kb inserted inversion including the *PML* gene located directly upstream of *GOLGA6B* (ogm[GRCh37]inv(15)(q24.1q24.1)(72,947,038_72,959,738)). In the wild-type situation, *PML* (chr15:74,287,014–74,340,155) is located upstream of *GOLGA6A* (chr15:74,362,198–74,374,891). After closer inspection of the OGM data, the whole 1.4 Mb region between *GOLGA6B* and *GOLGA6A* (ogm[GRCh37]inv(15)(q24.1q24.1)(72,959,741_74,362,190)) is inverted, and the complete *PML* is included in the inversion (supplementary information 5, Fig. 1a). The breakpoints are located within or nearby *GOLGA6A* and *GOLGA6B*, which have the same OGM label pattern. *GOLGA6A* and *GOLGA6B* are duplicons with 99.7% similarity. The uniquely mapped Nanopore reads became non-unique at the *GOLGA6B* locus. These reads are also non-uniquely mapping to the *GOLGA6A* locus (recognized by the read-ID), (supplementary information 5, Fig. 1b).

We identified forward-orientated (mutant) and reverse-orientated (wild-type) adapters at the CRISPR cutting sites (supplementary information 5, Fig. 1c). This means that the CRISPR cutting sites were located within the inverted region of the mutant allele, which does confirm the heterozygous inversion. The CRISPRs were designed in the wrong orientation because we initially misjudged the OGM aberration. As a result, we could not redefine the breakpoints. This inversion has not been described in the context of leukemia**.**

## Discussion

In this proof-of-principle study, we show that Cas9-directed LRS can be used to characterize low resolution areas of OGM at single-basepair-level to improve the interpretation of aberrations. Cas9-directed LRS confirmed several different types of OGM aberrations including translocations, deletions, insertions and inversions. In total we detected 23 OGM-aberrations in 18 BMAs. Of these aberrations, we confirmed 5 aberrations with Cas9-directed LRS because the resolution of the SOC methods was too low while we redefined the breakpoints of 5 other OGM-aberrations with insufficient resolution. We show that the actual breakpoints of the OGM calls are located between 0.2 and 5.5 kb of the OGM-estimated breakpoints, which supports previous reports.

This proof-of-principle study confirms that OGM is very reliable. Other studies (e.g.,^7,8^) did not attempt to confirm OGM findings below the resolution of the SOC methods, whereas we could also confirm such aberrations. Because of this, we expect that confirmation of OGM calls of known aberrations with sufficient resolution will not be needed after standard validation procedures in laboratories that want to implement this technology in their workflow. OGM is a developing technique that will unveil also new potentially clinically relevant aberrations, and these require validation, it will in such cases be useful to have Cas9-directed LRS available for confirmation. This approach needs extensive further validation, in particular because our study is small and implementation of a new diagnostic workflow needs a prolonged period of validation of variants.

Our approach allowed redefinition of a heterozygous 1.2 kb deletion in a 42 kb region (17p13.1) that includes *TP53* that fell between two OGM labels. Cas9-directed LRS showed that *TP53* is located upstream of the deletion. Another example was an uncertain OGM breakpoint of a translocation between chromosome 14 and 17 (q32.33; q25.3) where it was uncertain whether *IGHG1* was included. Cas9-directed LRS redefined the breakpoint to 88 kb upstream of *IGHG1.* These two examples demonstrate the relevance of using a high-resolution method like Cas9-directed LRS to follow-up OGM findings to determine the involvement of genes in a structural aberration. In germline genetic diagnostics but also in leukemia this might be of utmost importance for clinical interpretation.

The coverage of the region of interest of Cas9-directed LRS depends on biological variation and technical variables as DNA-quality, crRNA efficiency, sample-prep performance and sequencing performance. Indeed, the number of reads covering the targeted aberration (1–25 reads) was variable and sometimes sub-optimal because of the piloting nature of this study. Still, the coverage of this targeted method is generally higher than a whole genome sequencing approach would yield. In theory, one read with the aberration is sufficient to confirm and redefine the aberration with Cas9-directed LRS. A high coverage would however make it easier to confirm and redefine aberrations, especially when the frequency of somatic cells with the aberration in the sample is low. In this pilot study, we detected a 283 kb *FOXP1* deletion (3% allele frequency) with OGM and confirmed and redefined this aberration with Cas9-directed LRS. If the expected frequency of the aberration is estimated to be relatively high based on the OGM data, it will be possible to decrease the sequencing costs by sequencing more than one sample on one flow-cell. Currently, we observe that we are capturing on average a higher number of reads with the aberration as we are getting better acquainted with Cas9-directed LRS.

The strength of Cas9-directed LRS is that it is an amplification-free method that can access repeat regions, GC-rich areas and homologous regions in contrast to methods requiring PCR, including short-read sequencing, long range PCR, and gap-PCR^21,22^. Additionally, these methods have a limited reach. For example, it would not have been possible to cover the whole 42 kb region between the two OGM-labels in case of the *TP53* region, because it was unknown where the aberration was located. For known and possibly returning yet characterized CNVs long range PCR can be used for confirmation as a possibly cheaper alternative to Cas9-directed LRS. Although short-read DNA-sequencing is an appropriate technique for genetic testing in certain leukemias^23^, it is not suitable to confirm all possible novel OGM aberrations (e.g., large inversions) because it is much more difficult to span the breakpoints with high confidence, in particular with aberrations of low frequency. Therefore, we suggest that Cas9-directed LRS is the most appropriate method for confirming aberrations detected by OGM. Adaptive sampling LRS, the other targeted option of ONT, is also able to redefine OGM breakpoints. However, we showed that a fixed starting-position is essential in certain circumstances to confirm inversions when the resolution of LRS is insufficient to distinguish e.g., gene from pseudogene or duplex genes (*GOLGA6A* and *GOLGA6B*). With adaptive sampling this feature is not available because fragmentation is random in contrast to Cas9-directed LRS. A similar approach is, in principle, possible using targeted Pacific Biosciences SMRT sequencing (Menlo Park, CA), but Cas9-directed LRS has the advantage that an amplification-free enrichment is already included in the workflow as opposed to the PacBio method which needs a separate enrichment. Since ONT is continually evolving, an already available alternative protocol will need to be used on the ONT platform (SQK-LSK109, ONT) with separate ordering of the components.

Any new diagnostic workflow should have a short turnaround time in order to provide the best clinical care in diagnostics. For this reason, it is important to have readily available validation options for disputed OGM calls. This approach may have prognostic value, as we showed for the 42 kb region that included *TP53* that fell between two OGM labels. Because we already know the genomic regions for which OGM has limited resolution, it is possible to design and validate guide-RNAs for known hotspots in advance. The turnaround time of Cas9-directed LRS can then be limited to 1 or 2 working day(s) after the OGM lab-procedure and analysis. This is similar to the turnaround time of FISH. The total turnaround time is about 7 days. A short turnaround time is desired for prognosis and treatment in leukemia diagnostics. In a situation where no guide-RNAs are readily available, it will take 1.5–2 weeks to design and order guide-RNAs. However, we expect that guide-RNAs can be stored for years, which will allow extensive libraries to be built up for validation purposes.

We and others have shown that OGM reveals novel germline and somatic structural abnormalities and more complex aberrations. Similar as to how for example NGS evolved, we expect that prediction of clinical significance of new variants will improve over time through growing knowledge and enriched variant databases, both of which can be accelerated by cooperation in (inter)national working groups. Although OGM is a method that accesses the whole genome, we are currently forced to limit the region of interest to regions known to be involved in leukemia due to the high number of variants of uncertain significance. In the future, extending the region of interest may provide additional information for better stratification of prognosis and treatment, with or without improved characterization of the aberrations with Cas9-directed LRS.

In conclusion, OGM in combination with Cas9-directed LRS is a workflow that can characterize (complex) aberrations down to single-basepair-level. This approach will improve the prediction of the clinical significance of OGM-identified aberrations. Although OGM is currently the only method that produces long-reads with the required coverage to resolve (somatic) complex rearrangements, in some cases, Cas9-directed LRS is needed to fill gaps where OGM resolution is insufficient.

### Supplementary Information

Supplementary Information.

## Data Availability

The data sets generated and/or analyzed in this study are available from the corresponding author upon reasonable request.

## Abbreviations

SNV: Single nucleotide variants
CNV: Copy number variation
OGM: Optical genome mapping
SOC: Standard of care
LRS: Long-read sequencing
BMA: Bone marrow aspirates
