# Supplementary material for: Cas9-directed long-read sequencing to resolve optical genome mapping findings in leukemia diagnostics
Source: Sci Rep. 2024 Apr 12;14:8508. doi: 10.1038/s41598-024-59092-6 (PMC11009395; doi:10.1038/s41598-024-59092-6)
Supplement: Supplementary file 1 — Supplementary Information. [file 41598_2024_59092_MOESM1_ESM.pdf]

Title manuscript: Cas9-directed long-read sequencing to resolve optical genome mapping findings in leukemia diagnostics

Supplemental Information 1: the OGM settings

Data-analysis for the *de novo* assembly and rare variant analysis (RVA) was optimized by comparing several filter settings (Supplemental Table 1). Settings with a variable number of aberrations are the positive predictive value (PPV) of the intra-fusions, the PPV of the inter-translocations, the presence of the variants in the control group and the masking of CNV-sensitive regions of the genome (Supplemental Table 2 and 3). As hematologic malignancies arise at all ages (Prog Tumor Res. 2016;43:87-100, Juliusson et. al.), we used the least stringent filtering of the aberrations in the control group. Some aberrations were filtered when masking the CNV-enriched regions or selecting the genes of interest using the Access software. For example, an inversion (3q25.33;q26.2) that includes *MECOM* was filtered out due to a bug in the Access v.1.7 software. Further, translocations and fusions without a gene of interest were filtered out even though these aberrations can be of clinical significance. As a consequence, we decided to use settings D (Supplemental Table 1) which had the lowest specificity and highest sensitivity for the final analysis. Inverted aberrations in the genes of interest and disease-causing aberrations without a gene of interest were filtered using a disease-specific BED-file (Supplemental Information 2). For this reason, disease-specific BED files were used to visualize the regions of interest, but not for initial filtering.

The final settings (settings D) are: insertion recommended (confidence 0) and minimum size 500 bp, deletion recommended (confidence 0) and minimum size 500 bp, inversion recommended (confidence 0.7) and minimum size 30 kbp, duplication recommended (confidence -1) and minimum size 30 kbp, intra-fusion recommended (confidence 0.05), and;

inter-translocation recommended (confidence 0.05). Detected events were subsequently filtered (less than or equal to 1%) using a database of approximately 300 individuals from 33 different ethnicities with no known genetic disorder available in Access v.1.7. Settings applied to all SV types were: SV-masking = all structural variants, VAF filter min = 0, VAF filter max = 1, SV self-molecule check = SV found in self-molecules, self-molecule count = 5, SV overlapping genes filter = all SVs. CNV filters were: CNV type = all, copy CNV confidence = recommended (0.99), CNV minimum size = 500 kbp, CNV masking filter = all. Aneuploidy filters were: aneuploidy type = all, aneuploidy confidence = recommended (0.95). AOH/LOH filters (*de-novo*) were: AOH/LOH minimum size = 0bp. GRCh37 hg19 (UCSC, Santa Cruz USA (UCSC)) was used as a reference genome.

Supplemental Table 1: Filter settings tested.

	Filterset A	Filterset B	Filterset C	Filterset D	**Filterset E**
Insertion	0	0	0	0	0
Deletion	0	0	0	0	0
Inversion	0.7	0.7	0.7	0.7	0.7
Duplication	-1	-1	-1	-1	-1
Intra-fusion	0.05	0.3*	0.05	0.05	0.3*
Inter-translocation	0.05	0.65*	0.05	0.05	0.65*
Control-group	0%	0%	1%	1%	1%
SV masking	all	all	all	all	all
Self-molecules count	5	5	5	5	5
SV chimeric score	show not failing	show not failing	show not failing	show not failing	show not failing
Copy Number Variants	0.99	0.99	0.99	0.99	0.99
Copy Number Variants masking	non-masked only	non-masked only	non-masked only	all	all
Aneuploidy	0.95	0.95	0.95	0.95	0.95

The settings of the several filters are shown in this table. The following positive predictive values are recommended in Bionano Access v.1.7: insertion/deletion = 0, inversion = 0.7, duplications = -1, intra-fusion = 0.05, inter-translocation = 0.05, CNV = 0.99, aneuploidy = 0.95. Recommended Bionano Access v.1.6 settings are the same except: intra-fusion = 0.3 and inter-translocation = 0.65. A control database was used for filtering. Variants with an occurrence of 0 or 1% in the database were considered for analysis. CNV-sensitive regions were masked with filter settings A, B and C. Other settings that were applied to all were: SV-masking = all, self-molecules-counts = 5, SC-chimeric-score-show-not-failing.

Supplemental Table 2: number of aberrations with the several filter-sets, *de-novo* pipeline

Type of aberration	Number of aberrations (Mean/SD)		Filterset A (Mean/SD)		Filterset B (Mean/SD)		Filterset C (Mean/SD)		Filterset D (Mean/SD)		Filterset E (Mean/SD)	
**Insertion**	7090.4	2588.4	8.3	2.9	8.3	2.9	15.2	4.3	15.2	4.3	15.2	4.3
**Deletion**	3538.3	1547.2	12.9	3.7	12.9	3.7	25.1	4.4	25.1	4.4	25.1	4.4
**Inversion**	207.2	24.1	0.2	0.5	0.2	0.5	0.4	0.7	0.4	0.7	0.4	0.7
**Duplication**	119.4	53.1	2.2	1.7	2.2	1.7	2.9	2.3	2.9	2.3	2.9	2.3
**Intra-Fusion**	99.0	57.0	4.7	5.7	1.0	1.2	5.7	5.7	5.7	5.7	1.2	1.4
**Inter-Translocation**	61.6	17.6	0.8	1.3	0.6	1.3	1.1	1.5	1.1	1.5	0.7	1.3
**AOH/LOH**	10.1	3.0	10.1	3.0	10.1	3.0	10.1	3.0	10.1	3.0	10.1	3.0
**CNV gain**	28.6	9.7	2.2	2.6	2.2	2.6	2.2	2.6	2.6	2.7	2.6	2.7
**CNV loss**	31.7	3.9	1.1	1.9	1.1	1.9	1.1	1.9	1.2	1.9	1.2	1.9
**aneuploidy Gain**	0.1	0.3	0.1	0.3	0.1	0.3	0.1	0.3	0.1	0.3	0.1	0.3
**aneuploidy Loss**	0.1	0.3	0.06	0.2	0.06	0.2	0.06	0.2	0.06	0.2	0.06	0.2

In the first column, the number of aberrations (mean, SD) after *de-novo* assembly are shown without filtering. In the other columns, the number of aberrations (mean, SD) are depicted for each filter set. The different settings are presented in table 1.

Supplemental Table 3: number of aberrations with the several filter-sets, RVA

Type of aberration	Number of aberrations (Mean/SD)		Filterset A (Mean/SD)		Filterset B (Mean/SD)		Filterset C (Mean/SD)		Filterset D (Mean/SD)		Filterset E (Mean/SD)	
**Insertion**	771.2	64.0	6.6	3.3	6.6	3.3	10.4	2.9	10.4	2.9	10.4	2.9
**Deletion**	751.5	34.2	14.1	5.3	14.1	5.3	21.4	5.4	21.4	5.4	21.4	5.4
**Inversion**	198.5	38.8	0.0	0.0	0.0	0.0	0.0	0.0	0.0	0.0	0.0	0.0
**Duplication**	237.0	99.8	2.1	1.1	2.1	1.1	3.6	1.8	3.6	1.8	3.6	1.8
**Intra-Fusion**	108.2	50.3	2.6	1.2	0.6	0.8	3.9	1.8	3.9	1.8	0.6	0.8
**Inter-Translocation**	58.9	27.7	0.5	1.4	0.1	0.3	0.6	1.4	0.6	1.4	0.1	0.3
**CNV gain**	25.3	8.5	1.5	1.8	1.5	1.8	1.5	1.8	1.6	2.1	1.6	2.1
**CNV loss**	37.8	12.6	1.1	2.3	1.1	2.3	1.1	2.3	1.1	2.3	1.1	2.3
**aneuploidy Gain**	0.2	0.4	0.2	0.4	0.2	0.4	0.2	0.4	0.2	0.4	0.2	0.4
**aneuploidy Loss**	0.2	0.4	0.1	0.3	0.1	0.3	0.1	0.3	0.1	0.3	0.1	0.3

In the first column, the number of aberrations (mean, SD) after rare variant analysis are shown without filtering. In the other columns, the number of aberrations (mean, SD) are depicted for each filter set. The different settings are presented in table 1.

Supplemental Information 2: BED files

Myeloid BED file

Myeloid BED file GRCh37 (hg19)			
Gene	**Chr**	**Chr Start**	**Chr End**
PRDM16	1	2985741	3355185
CSF3R	1	36931643	36948915
MPL	1	43803474	43820135
RBM15	1	110881944	110889303
NRAS	1	115247084	115259515
DNMT3A	2	25455829	25565459
SF3B1	2	198256697	198299771
IDH1	2	209100952	209119806
RASSF1	3	50367216	50378367
GATA2	3	128198264	128212030
MECOM	3	168801286	169381563
FIP1L1	4	54243819	54326103
CHIC2	4	54875957	54930815
PDGFRA	4	55095263	55164412
KIT	4	55524094	55606881
TET2	4	106067841	106200960
PDGFRB	5	149493401	149535422
RPS14	5	149823791	149829319
RANBP17	5	170288895	170727019
NPM1	5	170814707	170837888
DEK	6	18224399	18264799
CUX1	7	101459183	101927250
EZH2	7	148504463	148581441
MNX1	7	156786744	156802129
MNX1	7	156797546	156803347
PCM1	8	17780365	17887457
FGFR1	8	38268655	38326352
KAT6A	8	41786996	41909505
RUNX1T1	8	92967194	93115454
JAK2	9	4985244	5128183
MLLT3	9	20341662	20622514
ABL1	9	133589267	133763062
NUP214	9	134000980	134110057
MLLT10	10	21823573	22032559
HRAS	11	532241	535550
NUP98	11	3696239	3819022
WT1	11	32409321	32457081
CCND1	11	69455872	69469242
PICALM	11	85668213	85780139
ZBTB16	11	113931287	114121397
KMT2A	11	118307204	118397539

CBL	11	119076985	119178859
ETV6	12	11802787	12048325
ETNK1	12	22778075	22843608
KRAS	12	25357722	25403854
PTPN11	12	112856535	112947717
NCOR2	12	124808956	125052010
FLT3	13	28577410	28674729
RB1	13	48877882	49056026
PML	15	74287013	74340155
IDH2	15	90627211	90645708
CREBBP	16	3775055	3930121
MYH11	16	15796991	15950887
FUS	16	31191430	31206192
CBFB	16	67063049	67134958
PRPF8	17	1553922	1588176
TP53	17	7571719	7590868
NF1	17	29421944	29704695
RARA	17	38465422	38513895
SRSF2	17	74730196	74733493
SETBP1	18	42260137	42648475
CALR	19	13049413	13055304
CEBPA	19	33790839	33793470
ASXL1	20	30946146	31027122
RUNX1	21	36160097	36421595
ERG	21	39739182	40033704
U2AF1	21	44513065	44527688
BCR	22	23522551	23660224
MKL1	22	40806291	41032690
ZRSR2	23	15808573	15841382
BCOR	23	39910498	40036582
STAG2	23	123094409	123236505

Mature B cell neoplasm BED file

**Mature B cell neoplasm BED file GRCh37 (hg19)**			
Gene	**Chr**	**Chr Start**	**Chr End**
FAF1	1	50906935	51425936
CDKN2C	1	51434366	51440309
BCL10	1	85731459	85742587
MTF2	1	93544792	93604638
TMED5	1	93615299	93646246
FAM46C	1	118148604	118171011
CKS1B	1	154947118	154951725
ACP1	2	264868	278282
MYCN	2	16080559	16087129

ALK	2	29415639	30144477
MSH2	2	47630205	47710367
BCL11A	2	60684328	60780633
REL	2	61108629	61155291
XPO1	2	61705068	61765418
CXCR4	2	136871918	136875725
SF3B1	2	198256697	198299771
MYD88	3	38179969	38184510
SETD2	3	47057897	47205467
CDC25A	3	48198667	48229801
ATRIP	3	48488113	48507708
FOXP1	3	71003864	71633140
PIK3A-PIK3CA	3	178866310	178952497
BCL6	3	187439164	187463513
TP63	3	189349215	189615068
FGFR3	4	1795039	1810599
WHSC1	4	1873123	1983934
NPM1	5	170814707	170837888
DUSP22	6	292056	351355
IRF4	6	391738	411443
CCND3	6	41902671	42016610
SEC63	6	108188959	108279482
FOXO3	6	108881025	109005971
MYB	6	135502452	135540311
POT1	7	124462439	124570037
BRAF	7	140433812	140624564
TRAIL-TNFRSF10A	8	23048969	23082680
TRIM35	8	27142403	27168834
MYC	8	128748314	128753680
PVT1	8	128806778	129113499
MAFA	8	144510229	144512602
NOTCH1	9	139388884	139440238
PTEN	10	89623194	89731687
PIK3A	10	98353068	98480279
CCND1	11	69455872	69469242
MRE11A	11	94150468	94227040
BIRC3	11	102188180	102210135
ATM	11	108093558	108239826
H2AFX	11	118964584	118966177
CCND2	12	4382901	4414522
RB1	13	48877882	49056026
DLEUregion	13	50456688	51417885
DLEU2	13	50556687	50699677
BCMS-DLEU	13	50975361	51205735
TGDS	13	95226307	95248529
MGA	15	41952609	42062141
IGH	16	31973408	31973499

MAF	16	79627744	79634622
PLCG2	16	81812898	81991899
TP53	17	7571719	7590868
CLTC	17	57697049	57774317
MALT1	18	56338617	56417371
BCL2	18	60790578	60986613
MAFB	20	39314516	39317876
PRAME	22	22890117	22901768
BTK	23	100604434	100641212

ALL BED file

**ALL BED file GRCh37 (hg19)**			
Gene	**Chr**	**Chr Start**	**Chr End**
TAL1	1	47681961	47698007
STIL	1	47715810	47779819
JAK1	1	65298905	65432187
MEF2D	1	156433512	156460391
PBX1	1	164528596	164821060
ABL2	1	179068461	179112224
FHIT	3	59735035	61237133
PDGFRA	4	55095263	55164412
AFF1	4	87856153	88062206
DUX4	4	190998875	191000255
IL3	5	131396346	131398896
CSF1R	5	149432853	149492935
PDGFRB	5	149493401	149535422
EBF1	5	158122922	158526788
TLX3	5	170736287	170739138
RUNX2	6	45296053	45518819
MYB	6	135502452	135540311
MLLT4-AS1	6	168224569	168227476
MLLT4	6	168227670	168372700
HOXA13	7	27236498	27239725
IKZF1	7	50344377	50472798
TRB	7	142197571	142198055
MYC	8	128748314	128753680
JAK2	9	4985244	5128183
MLLT3	9	20344967	20622514
CDKN2A	9	21967750	21994490
CDKN2B	9	22002901	22009312
PAX5	9	36833271	37034476
ABL1	9	133589267	133763062
NUP214	9	134000980	134109091

MLLT10	10	21823573	22032559
PTEN	10	89623194	89731687
BLNK	10	97951454	98031333
TLX1	10	102891060	102897546
ADD3	10	111756107	111895323
NUP98	11	3696239	3819022
LMO1	11	8245850	8290182
LMO2	11	33880122	33913836
RAG1	11	36589563	36601310
RAG2	11	36613492	36619829
KMT2A	11	118307204	118397539
ZNF384	12	6775642	6798738
ETV6	12	11802787	12048325
BTG1	12	92534053	92539673
RB1	13	48877882	49056026
TRA	14	22180545	22181065
TRD	14	22564301	22564896
BCL11B	14	99635624	99737822
PAR1	15	25380788	25383200
IGH	16	31973408	31973499
NF1	17	29421944	29704695
IKZF3	17	37913967	38020441
HLF	17	53342320	53402426
TCF4	18	52889562	53303224
TCF3	19	1609291	1652326
MLLT1	19	6210391	6279959
EPOR	19	11487880	11495018
JAK3	19	17935592	17958841
ELL	19	18553472	18632937
RUNX1	21	36160097	36421595
ERG	21	39739182	40033704
TMPRSS2	21	42836477	42880085
BCR	22	23522551	23660224
CRLF2	23	1314886	1331616
CSF2RA	23	1387692	1428828
IL3RA	23	1455508	1501582
P2RY8	23	1581465	1656037

Supplemental Information 3: OGM procedure, OGM-detected aberrations and confirmation of these aberrations with the SOC methods

After collection of the samples, 15 µl stabilizing buffer (Bionano) was added to 1 ml of bone marrow aspirate (BMA). Samples were stored at -80°C until transported on dry ice to a Bionano certified service provider lab (INRAe, Clermont-Ferrand, France), where the OGM procedure was performed following the manufacturer's instructions. For each sample, 1 mL of BMA was used to purify ultra-high molecular weight (UHMW) DNA using the Bionano Prep SP BMA DNA Isolation kit. Molecules were labeled with the DLS (Direct Label and Stain) DNA Labeling Kit. Saphyr chips were run to reach a minimum yield of 1300 Gb. The *de novo* assembly, RVA and variant annotation pipeline were executed on Solve v.3.7 (Bionano). The *de novo* assembly was run with a coverage of ~200x and the RVA with ~400x mapped reads. Reporting and direct visualization of structural variants was done on Access v.1.7 (Bionano). Filtering of the structural variants was optimized by testing several filter settings. OGM was successfully executed for all 18 bone marrow samples, and 23 aberrations were detected in the regions of interest. In total, 13/23 OGM findings could be confirmed with the SOC methods, and no SOC-detected aberrations were missed by OGM (supplemental table 4).

Supplemental Table 4: OGM-detected aberrations

ID	Referral reason	OGM (splitted per aberration)	*De novo* / RVA / Both	Reason not detected	SOC	Identified with SOC
BMA1	AML/MDS/MPN	ogm[GRCh37]1q21.1q21.1(144452084_249237532)x2~3	both		Karyotyping	46,XY,der(13)t(1;13)(q12;p11)
		ogm[GRCh37]17p13.1(7,545,377_7,588,071)x1	*de novo*	RVA only aberrations >5kb	Could not be confirmed	-
BMA2	CML	ogm[GRCh37]t(9;22)(q34;q11.2)(133619362_23603312)	both		Karyotyping	46,XY,t(9;22)(q34;q11.2)
BMA3	AML/MDS	ogm[GRCh37]17p13.1(7,545,377_7,588,071)x1	*de novo*	RVA only aberrations >5kb	Could not be confirmed	-
BMA4	MDS/MPN	ogm[GRCh37](X,1-22)x2 (no aberration)	both		SNP-arrays	arr(X,1-22)x2
BMA5	MDS	ogm[GRCh37](X,Y)x1,(1-22)x2 (no aberration)	both		SNP-arrays	arr(X,Y)x1,(1-22)x2
BMA6	MDS	ogm[GRCh37]17p13.1(7,545,377_7,588,071)x1	*de novo*	RVA only aberrations >5kb	Could not be confirmed	-
BMA7	MDS/MF	ogm[GRCh37]inv(3)(q25.33q26.2)(160,014,744_168,882,939)	both		Could not be confirmed	-
		ogm[GRCh37]3q25.33(159,902,689-160,014,744)x1	both		Could not be confirmed	-
		ogm[GRCh37]3q26.2(168,882,939-168,907,480)x1	both		Could not be confirmed	-
BMA8	CML	ogm[GRCh37]t(9;22)(q34;q11.2)	both		Karyotyping	46,XX,t(9;22)(q34;q11.2)
		ogm[GRCh]22q11.23;q12.1)(23,734,214;29,424,129)x1	both		SNP-arrays	arr[GRCh37] 22q11.23q12.1(23632968_29447635)x1
BMA9	MDS	ogm[GRCh37]chr21x3	both		SNP-arrays	arr(21)x3
		ogm[GRCh37]inv(15)(q24.1q24.1)(72,959,741_74,362,190)	both		Could not be confirmed	-
		ogm[GRCh37]3p13(71086423_71375386)x1	RVA		Could not be confirmed	-
BMA10	MDS/MPN	ogm[GRCh37]9p24.3p13.3(1_35889394)x2 hmz	*de novo*	RVA not able to detect hmz	SNP-arrays	arr[GRCh37] 9p24.3p13.3(1_35889394)x2 hmz,
		ogm[GRCh37]14q11.2q12(20421719_32361050)x1	both		SNP-arrays	14q11.2q12(20421719_32361050)x1
BMA11	ALL/CLL	ogm[GRCh37](X,Y)x1,(1-22)x2 (no aberration)	both		Karyotyping	46,XY
BMA12	(B)-CLL	ogm[GRCh37]chr12x3	both		SNP-arrays	arr(12)x3
BMA13	MDS	ogm[GRCh37] 20q11.22q13.13(34259243_49502193)x1	both		SNP-arrays	arr[GRCh37] 20q11.22q13.13(34259243_49502193)x1
BMA14	?	ogm[GRCh37](X,Y)x1,(1-22)x2 (no aberration)	both		Karyotyping	46,XY
BMA15	MDS/AML	ogm[GRCh37] fus(5;5)(q14.2;q33.3)	RVA	5% aberrant cells non-cultured	Karyotyping, SNP-arrays	46,XX,5q-[5]/46,XX[5] (only OGM RVA)
BMA16	HCL (CLL)	ogm[GRCh37](X)x1,(1-22)x2	both		Karyotyping	45,X,-Y[13]/46,XY[7]
		ogm[GRCh37]8p23.3p22(11805_15540773)x1~2	both		SNP-arrays	arr[GRCh37] (Y)x0,8p23.3p22(1_15547178)x1, 8q13.2q24.3(69538276_146364022)x3
		ogm[GRCh37]fus(8;8)(p22;q13.2)	both		Karyotyping (1:250 metaphases)	fus(8;8)(p22;q13.2),t(14;17)(q32.33;q25.3)
		ogm[GRCh37]t(14;17)(q32.22;q25.3) (106,249,815;80,915,618)	both	Disputable OGM breakpoint	Could not be confirmed	-
BMA17	MDS	OGM[GRCh37]ins(12p13.2)(11,889,007_11,895,594)	*de novo*	RVA only aberrations >5kb	Could not be confirmed	-
BMA18	AML	ogm[GRCh37](X,(1-22)x2	both			46,XX[20]

All OGM aberrations detected in a selected group of leukemia patients are depicted in this table. The table also shows if the OGM aberrations are confirmed with the SOC methods.

Abbreviations: ID = sample identification, AML = acute myeloid leukemia, MDS = myelodysplastic syndrome, MPN = myeloproliferative neoplasms, CML = chronic myeloid leukemia, MF = myelofibrosis, ALL = acute lymphoblastic leukemia , CLL = chronic lymphoblastic

leukemia, (B)-CLL = B cell acute lymphoblastic leukemia, HCL = hairy cell leukemia, SOC = standard-of-care, OGM = optical genome mapping.

A homozygous region (ogm[GRCh37]9p24.3p13.3(1_35889394)x2 hmz) in sample BMA10 with referral reason myelodysplastic syndrome / myeloproliferative neoplasms was only detected with the *de novo* pipeline because the RVA pipeline cannot detect homozygous regions (Bionano, 30110 Rev K).

A 1.2 kb heterozygous 17p13.1 deletion (ogm[GRCh37]17p13.1(7,545,377_7,588,071)x1) in a 42 kb region that includes *TP53* in samples BMA1, BMA3 and BMA6 and a 1.4 kb heterozygous 12p13.2 insertion in a 6.6 kb region that includes *ETV6* (OGM[GRCh37]ins(12p13.2)(11,889,007_11,895,594)) in sample BMA17 with referral reason myelodysplastic syndrome were only detected with the *de novo* pipeline. The aberrations in BMA1, BMA3, BMA6 and BMA17 were below the resolution of the RVA pipeline (5 kb, Bionano, 30110 Rev K).

A fusion of chromosome 5 (ogm[GRCh37] fus(5;5)(q14.2;q33.3)) in patient BMA15, with referral reason myelodysplastic syndrome / acute myeloid leukemia was only detected with the RVA pipeline. Karyotyping of the aberration identified it as a chromosome 5q deletion in 5 of 10 metaphases. Using SNP-array on non-cultured cells, the estimated percentage of aberrant cells was 5%, which is below the threshold of the *de novo* pipeline (Bionano, 30110 Rev K).

In patient BMA9, with referral reason myelodysplastic syndrome, a 288 kb deletion on chromosome 3 (ogm[GRCh37]3p13(71086423_71375386)x1) with an allele frequency of 3% was only detected with the RVA pipeline. An allele frequency of 3% is below the resolution of the *de novo* pipeline (Bionano, 30110 Rev K). All other aberrations were detected with both pipelines.

In patient BMA16, with referral reason hairy cell leukemia, OGM identified a loss of the p-arm of chromosome 8p23.3p22 (ogm[GRCh37]8p23.3p22(11805_15540773)x1~2) and

a chromosome 8 fusion (ogm[GRCh37]fus(8;8)(p22;q13.2)) (table). SNP-array detected a gain and loss of chromosome 8 (arr[GRCh37](Y)x0,8p23.3p22(1_15547178)x1, 8q13.2q24.3(69538276_146364022)x3), and we performed karyotyping to confirm the fusion. The fusion was detected in only 1 of 250 metaphases, probably due to the loss of aberrant mature B cells in the culturing procedure (1). The clinical significance of the fusion is unknown (2,3).

In patient BMA1, with referral reason acute myeloid leukemia / myelodysplastic syndrome / myeloproliferative neoplasms, karyotyping confirmed a gain of 1q21.1q21.1 (ogm[GRCh37]1q21.1q21.1(144452084_249237532)x2~3) as a 46,XY,der(13)t(1;13)(q12;p11) with karyotyping.

References Supplemental Information 3

1. Troussard X, Cornet E. Hairy cell leukemia 2018: update on diagnosis, risk-stratification, and treatment. Am J Hematol. 2017;12:1382-1390.

2. The 1000 Genomes Project Consortium. A global reference for human genetic variation. Nature 2015;526(7571):68-74.

3. Edelmann J, Holzmann K, Miller F, Winkler D, Bühler A, Zenz T et al. High-resolution genomic profiling of chronic lymphocytic leukemia reveals new recurrent genomic alterations. Blood 2012;120(24):4783-4794.

Supplemental Information 4

Supplemental Table 5: Crispr locations.

ID	Additional putative OGM aberration	Gene	crRNA GRCh37 hg19
**BMA1**	OGM[GRCh37]17p13.1(7,545,377_7,588,071)x1	*TP53*	chr17:7,543,974-7,543,993 +
**BMA3**	OGM[GRCh37]17p13.1(7,545,377_7,588,071)x1	*TP53*	chr17:7,559,625-7,559,644 -
**BMA6**	OGM[GRCh37]17p13.1(7,545,377_7,588,071)x1	*TP53*	chr17:7,556,557-7,556,576 +
			chr17:7,576,982-7,577,001 -
			chr17:7,572,406-7,572,425 +
			chr17:7,589,483-7,589,502 -
**BMA9**	OGM[GRCh37]3p13(71,086,423_71,375,386)x1	*FOXP1*	chr3:71,082,359-71,082,378 +
			chr3:71,101,049-71,101,068 –
			chr3:71,082,955-71,082,974 +
			chr3:71,101,401-71,101,420 –
			chr3:71,371,938-71,371,957 +
			chr3:71,384,613-71,384,632 –
			chr3:71,368,075-71,368,094 +
			chr3:71,382,344-71,382,363 –
**BMA17**	OGM[GRCh37]ins(12p13.2)(11,889,007_11,895,594)	*ETV6*	chr12:11,888,244-11,888,263 +
			chr12:11,902,197-11,902,216 -
			chr12:11883363-11883382 +
			chr12:11910042-11910061 -
**BMA16**	OGM[GRCh37]t(14;17)(q32.22;q25.3)<br>(106,249,815;80,915,618)	*IGHG1*	chr17:80967365-80967384 +
			chr17:80996130-80996149 +
			chr14:106126211-106126230 -
			chr14:106100173-106100192 -
			chr14:106083832-106083851 -
**BMA7**	OGM[GRCh37]inv(3)(q25.33;q26.2)(160,014,744_168,882,939<br>)	*MECOM*	chr3:159898486-159898505 +
	OGM[GRCh37]3q25.33(159,902,689-160,014,744)x1		chr3:160032530-160032549 -
	OGM[GRCh37]3q26.2(168,882,939-168,907,480)x1		
**BMA9**	OGM[GRCh37]inv(15)(q24.1;q24.1)(72,959,741_74,362,190)	*PML*	chr15:72975831-72975850 -
			chr15:72966403-72966422 -

For each additional OGM-detected putative aberration, this table lists the sample ID, the genes involved and the GRCh37 coordinates of the crRNA enzymes.

Supplementary Information 5: Images Bionano Access and IGV-browser

Figure 1: BMA9, ogm[GRCh37]inv(15)(q24.1q24.1)(72,959,741_74,362,190)
OGM detected an inverted 1.4 Mb region between *GOLGA6A* and *GOLGA6B* which includes *PML* (figure 1a). The breakpoints are located within or nearby *GOLGA6A* and *GOLGA6B*, which are duplicons with 99.7% similarity. ONT-reads could map to both genes (figure 1b, same read-ID) and consequently we were not able to redefine the OGM-breakpoints. However, forward (mutant) and reverse (wild-type) orientated adapters were identified at the CRISPR sites (figure 1c), which confirms the heterozygous inversion.

Figure 1a

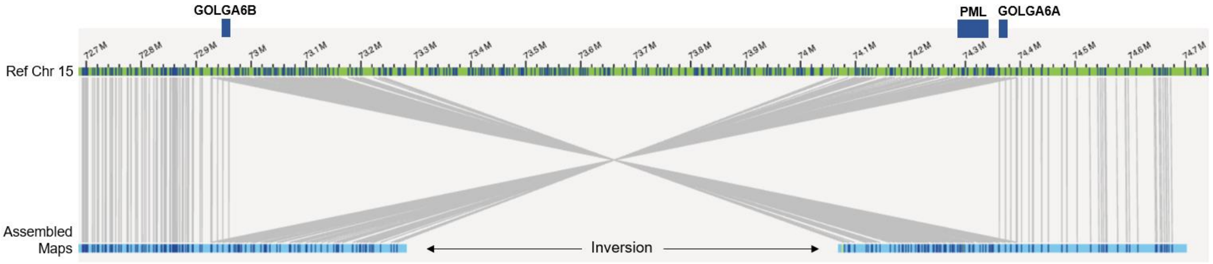

Figure 1b

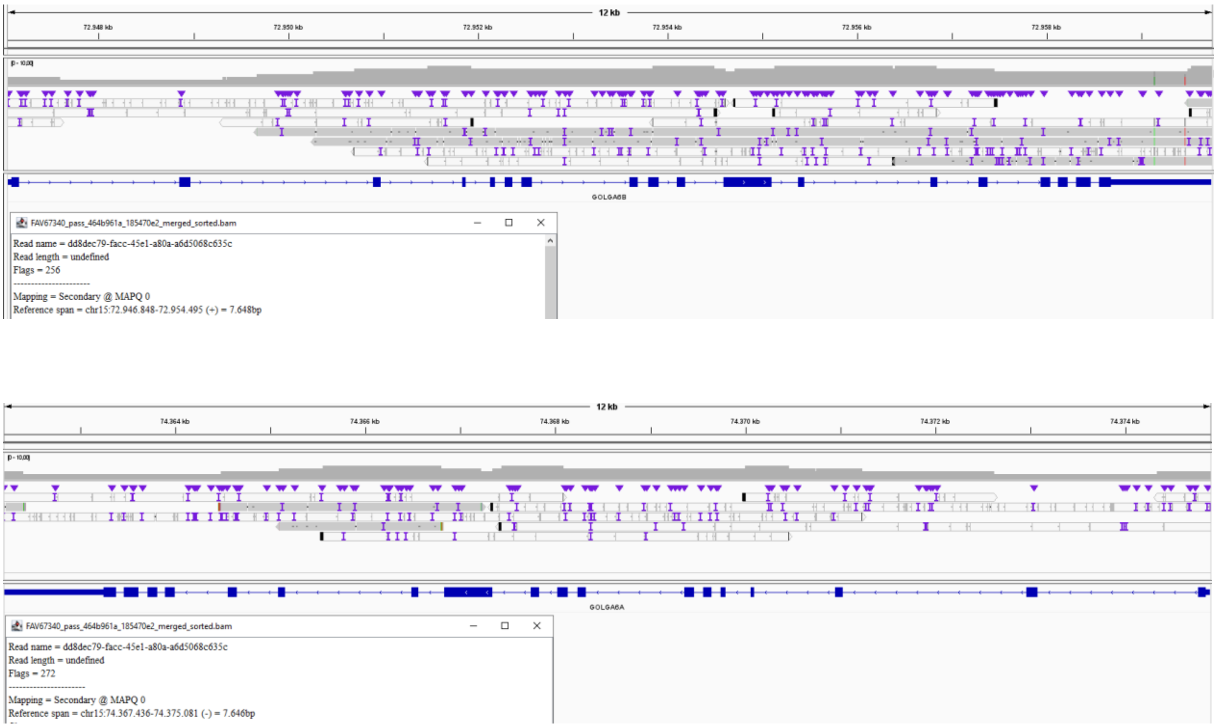

Figure 1c

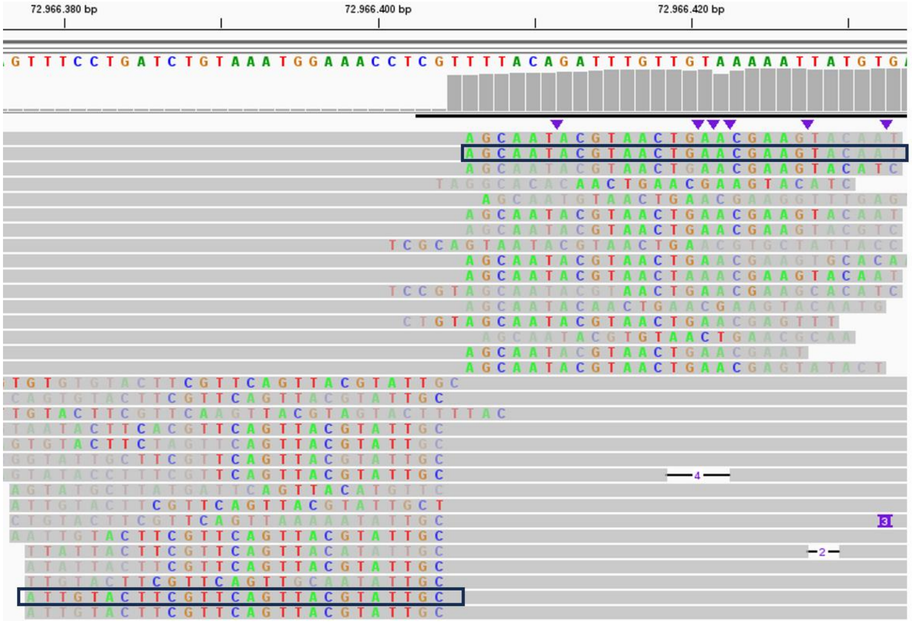

Figure 2: BMA17, ogm[GRCh37]ins(12p13.2)(11,889,007_11,895,594)

OGM detected a heterozygous 1.4 kb insertion in a 6.6 kb region located between two OGM labels that includes *ETV6* (chr12:11,802,788-12,048,325) (figure 2a). Cas9-directed LRS redefined the insertion to 1.4 kb (figure 2b). This 1.4 kb insertion is 99% similar to region chr12:11,892,052-11,893,440 and is inserted in intron 1 (chr12:11,892,830-11,894,194) of the NM_001987 transcript of *ETV6*.

Figure 2a

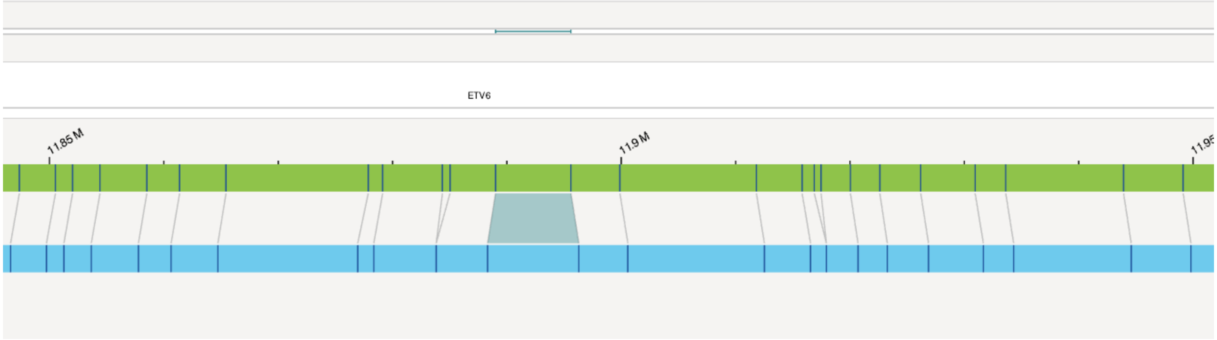

Figure 2b

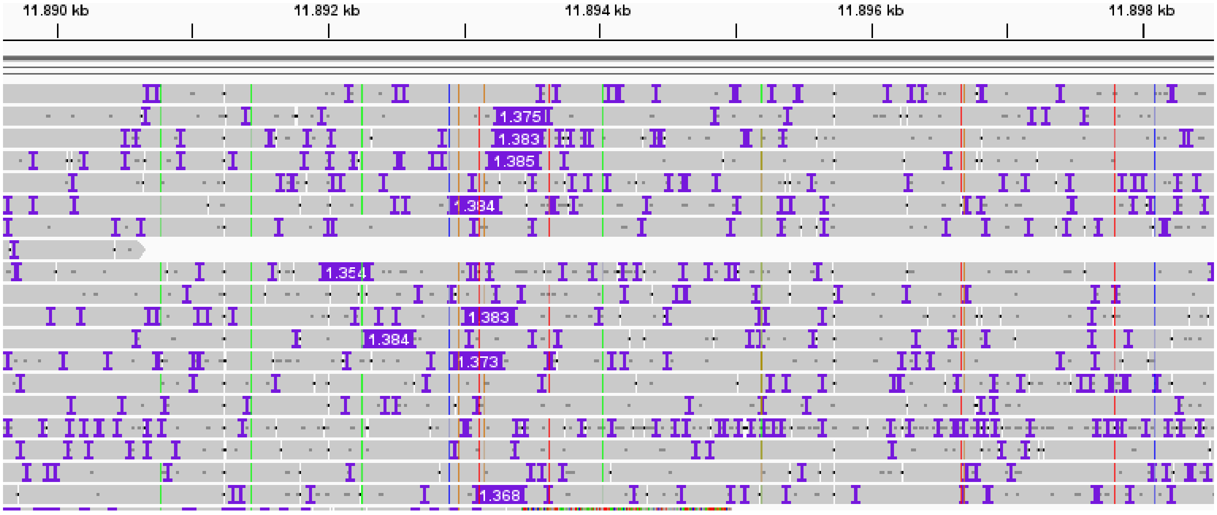

Figure 3: BMA9, ogm[GRCh37]3p13(71,086,423_71,375,386)x1

OGM detected a heterozygous 289 kb deletion that includes *FOXP1* (figure 3a). This aberration, which has an allele frequency of 3%, was only detected with the rare variant pipeline (Supplemental Information 3). Cas9-directed LRS redefined the aberration to a 283 kb (71,087,137-71,370,212) deletion of intron 7 through intron 11 of *FOXP1* (figure 3b, same read-ID maps to two locations in the alignment).

<u>Figure 3a</u>

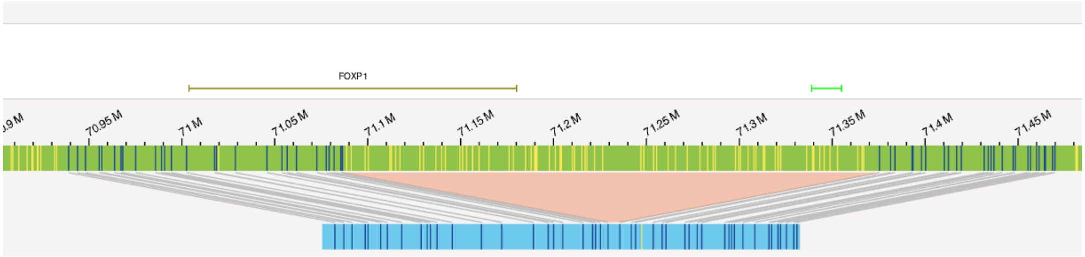

Figure 3b

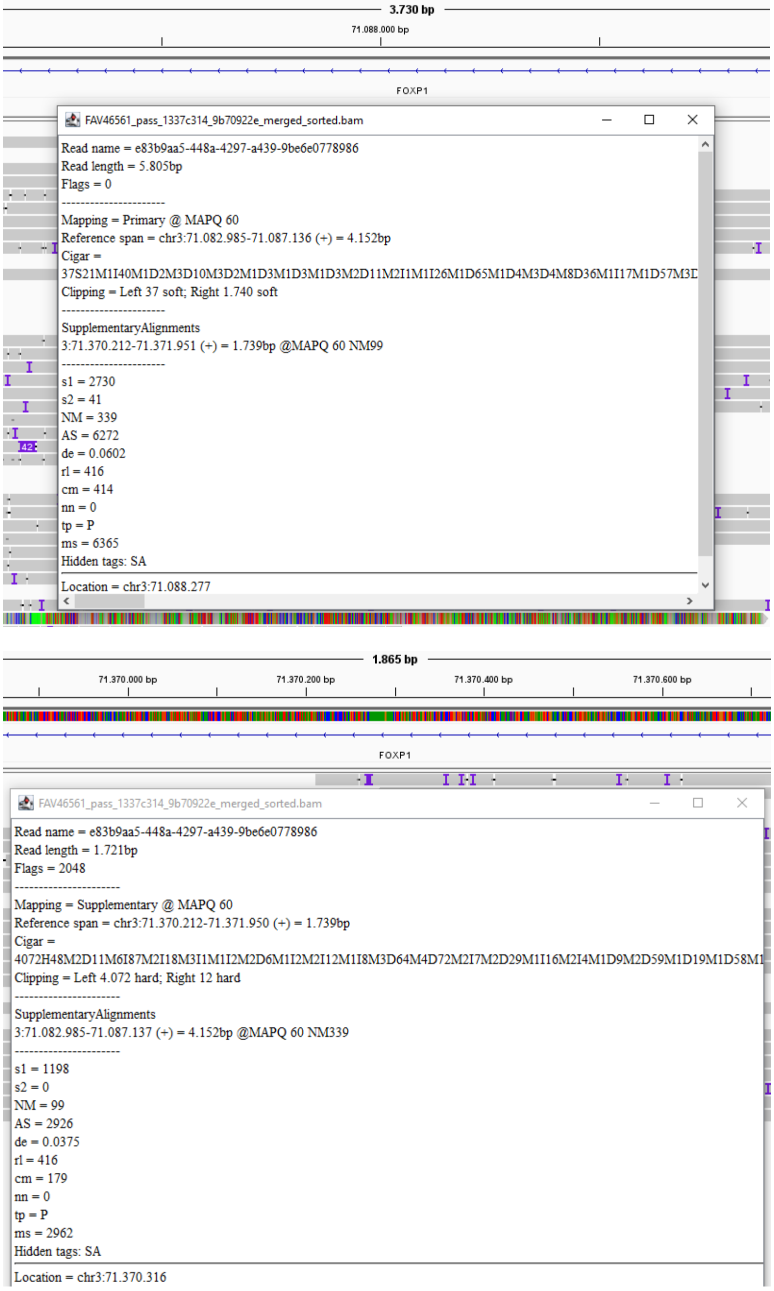
3.730 bp
71.088.000 bp
FOXP1
FAV46561_pass_1337c314_9b70922e_merged_sorted.bam
Read name = e83b9aa5-448a-4297-a439-9be6e0778986
Read length = 5.805bp
Flags = 0
Mapping = Primary @ MAPQ 60
Reference span = chr3:71.082.985-71.087.136 (+) = 4.152bp
Cigar =
37S21M1I40M1D2M3D10M3D2M1D3M1D3M1D3M2D11M2I1M1I26M1D65M1D4M3D4M8D36M1I17M1D57M3D
Clipping = Left 37 soft; Right 1.740 soft
SupplementaryAlignments
3:71.370.212-71.371.951 (+) = 1.739bp @MAPQ 60 NM99
s1 = 2730
s2 = 41
NM = 339
AS = 6272
de = 0.0602
rl = 416
cm = 414
nn = 0
tp = P
ms = 6365
Hidden tags: SA
Location = chr3:71.088.277
1.865 bp
71.370.000 bp
71.370.200 bp
71.370.400 bp
71.370.600 bp
FOXP1
FAV46561_pass_1337c314_9b70922e_merged_sorted.bam
Read name = e83b9aa5-448a-4297-a439-9be6e0778986
Read length = 1.721bp
Flags = 2048
Mapping = Supplementary @ MAPQ 60
Reference span = chr3:71.370.212-71.371.950 (+) = 1.739bp
Cigar =
4072H48M2D11M6I87M2I18M3I1M1I2M2D6M1I2M2I12M1I8M3D64M4D72M2I7M2D29M1I16M2I4M1D9M2D59M1D19M1D58M1
Clipping = Left 4.072 hard; Right 12 hard
SupplementaryAlignments
3:71.082.985-71.087.137 (+) = 4.152bp @MAPQ 60 NM339
s1 = 1198
s2 = 0
NM = 99
AS = 2926
de = 0.0375
rl = 416
cm = 179
nn = 0
tp = P
ms = 2962
Hidden tags: SA
Location = chr3:71.370.316
